# Why are somatic diseases in bipolar disorder insufficiently treated?

**DOI:** 10.1186/s40345-019-0147-y

**Published:** 2019-05-05

**Authors:** René Ernst Nielsen, Pirathiv Kugathasan, Sune Straszek, Svend Eggert Jensen, Rasmus W. Licht

**Affiliations:** 1 Department of Clinical Medicine, Aalborg University, Aalborg, Denmark; 2 Department of Psychiatry, Aalborg University Hospital, Mølleparkvej 10, 9000 Aalborg, Denmark; 3 Department of Cardiology, Aalborg University Hospital, Aalborg, Denmark

**Keywords:** Bipolar disorder, Mortality, Morbidity, Somatic illness

## Abstract

**Background:**

Somatic diseases, including cardiovascular, respiratory, and cancer diseases, are the main contributors to a shortened life expectancy of 10–20 years in patients with bipolar disorder as compared to the general population. In the general population an increase in survival has been observed over the last decades, primarily due to the advances in primary prophylaxis, medical treatment and progress in early detection and monitoring of somatic diseases. In this narrative review, we discuss the existing literature on treatment and outcomes of cardiovascular, respiratory, and cancer diseases in patients with bipolar disorder, and put this in the context of findings in studies on patients diagnosed with other severe mental disorders.

**Main body:**

The existing literature suggests that patients with bipolar disorder receive fewer or delayed medical interventions, when admitted with severe somatic diseases, compared to those not diagnosed with bipolar disorder. Cardiovascular disease is the most investigated disease regarding outcomes in patients with severe mental illness, and novel findings indicate that the increased mortality following cardiac events in these patients can be reduced if they are intensively treated with secondary prophylactic cardiac intervention. Elderly patients diagnosed with mental disorders and cancer experience a delay in receiving specific cancer treatment. No studies have investigated treatment outcomes in patients with severe mental disease and respiratory diseases.

**Conclusion:**

It is surprising and of major concern that patients with bipolar disorder have not benefitted from the significant improvement that has taken place over time over time of somatic treatments in general, especially in countries with equal and free access to healthcare services. Therefore, no matter whether this situation is a result of a negative attitude from health care providers to patients with mental illness, the result of the patient’s lack of awareness of their physical illness or the results of other factors, further attention including research on developing strategies for improving the management of somatic diseases in patients with bipolar disorder is needed.

## Background

Data from both primary and secondary care have shown that bipolar disorder is associated with an increased relative mortality rate over time as compared to the general population (Hoang et al. 2011; Hayes et al. 2015, 2017; John et al. 2018; Staudt Hansen et al. 2018); some data even suggest that an increased life expectancy in the general population is the primary driver, more than a decreased life expectancy in patients with bipolar disorder (Hayes et al. 2017). A recent meta-analysis indicated that for patients diagnosed with bipolar disorder, the standardized mortality ratio (SMR) due to suicide was 13–16 times higher, compared to the SMR of 1.7 for natural causes of death as compared to the general population (Hayes et al. 2015). Suicide often occurs early in the course of the bipolar disorder (Plans et al. 2019), and therefore results in large percentage contribution to the reduced overall life expectancy, despite the relatively low overall number of deaths from this cause (see below) (Kessing et al. 2015; Jayatilleke et al. 2017). The percentage contribution of suicide to the reduced overall life expectancy as compared to the general population was approximately 13.5%, whereas cardiovascular disease contributed 17.4% and 22.0% to the reduced overall life expectancy in males and females, respectively (Jayatilleke et al. 2017). As to the contributions to the number of total deaths, Westman et al. showed that approximately one in three patients with bipolar disorder dies from cardiovascular disease, with almost half dying from other somatic diseases (Westman et al. 2013). Likewise, Jayatilleke et al. showed that the main causes of death for patients diagnosed with severe mental illness (SMI), including bipolar disorder, in general were cardiovascular disease (23.9% of males and 17.6% of females), cancer (13.5% of males and 19.9% of females) and respiratory diseases (16.2% of males and 15.4% of females), with 6.7% of males and 4.4% of females with SMI having suicide recorded as cause of death (Jayatilleke et al. 2017). These findings are similar to the findings in older studies which in patients diagnosed with bipolar disorder mainly showed an increased relative mortality rate caused by cardiovascular and respiratory disease with some studies showing an increased and others showing no increased risk of cancer as specific cause of death (Roshanaei-Moghaddam and Katon 2009) with the main causes of death being cardiovascular disease, cancer and infection in the general population (Nielsen et al. 2013).

In the present review, we describe the findings regarding treatment and mortality of patients diagnosed with bipolar disorder treated for either cardiovascular disease, respiratory disease or cancer, as well as discuss the findings in relation to studies on non-bipolar patients diagnosed with SMI when relevant, as these somatic comorbidities are the main causes of death in patients with bipolar disorder as outlined above. A major limitation in all the studies including meta-analyses selected for review is the lack of reporting how the various somatic diseases have been diagnosed. However, we assume that most cases of somatic disease are diagnosed by clinicians, taking patient report, clinical examination and laboratory tests into account; at least this is how diagnoses entered into administrative registers like the Danish nationwide registers, are obtained (Kessing et al. 2015).

## Cardiovascular disease

Cardiovascular risk factors like smoking, obesity, hypertension, increased cholesterol or diabetes are twice as common in patients diagnosed with bipolar disorder as compared to the background population (Birkenaes et al. 2007; Hsu et al. 2015), with a possible earlier onset of these risk factors in bipolar patients (Birkenaes et al. 2007; Goldstein et al. 2009; Chien et al. 2013; Hsu et al. 2015) resulting in an increased all-cause mortality as well as cardiovascular-specific mortality as compared to the general population (Laursen et al. 2013; Correll et al. 2017; Staudt Hansen et al. 2018). The cluster of the risk factors hypertension, hyperglycemia, excess abdominal fat and abnormal cholesterol/triglycerides is termed the metabolic syndrome. The rate of mortality as caused by cardiovascular disease is approximately doubled in patients with bipolar disorder as compared to the background population, with a decreased life expectancy of 10 to 15 years (Laursen et al. 2013). Previous intervention studies in patients with SMI have primarily focused on health behavior change, as the most common cardiovascular risk factors, e.g. smoking, sedentary lifestyle and obesity, are modifiable (Kilbourne et al. 2013; Speyer et al. 2016; Ashdown-Franks et al. 2018). Generally, these studies have been unable to show an effect of the interventions tested when compared to no intervention (Kilbourne et al. 2012, 2013; Speyer et al. 2016; Ashdown-Franks et al. 2018). However, a recent meta-analysis showed short term effect of lifestyle interventions in patients with SMI on cardiovascular risk factors (Singh et al. 2018; Vancampfort et al. 2019). Furthermore, studies addressing medical conditions and health-risk behavior in patients diagnosed with schizophrenia and bipolar disorder have shown effect of both metformin and behavioral interventions in reducing obesity, cholesterol, glucose and insulin resistance, as well as effect of bupropion and varenicline in reducing tobacco smoking (McGinty et al. 2015).

Primary prophylactic treatment, e.g. preventing or increasing resistance to a disease that has not occurred, in the general population is most often driven by cardiovascular risk assessment, e.g. Framingham Heart Score, which however might not predict risk of cardiovascular events in patients with SMI. Therefore, models specifically developed to account for the increased cardiovascular risk in patients with SMI should be implemented (Osborn et al. 2015). A newer study on patients diagnosed with schizophrenia has shown a dose-dependent effect of secondary prophylactic treatment, e.g. preventing the recurrence of a disease that has already occurred, after myocardial infarction (Kugathasan et al. 2018a, b); and moreover that with increasing treatment intensity, the patients had a decrease in excess mortality relative to the population of patients with myocardial infarction, not diagnosed with schizophrenia, until no excess mortality was found with the most intense treatment investigated. Previously, Laursen et al. showed a lowered rate of invasive cardiac procedures in patients diagnosed with schizophrenia, schizo-affective disorder or bipolar disorder as compared to patients not diagnosed with a psychiatric condition (Laursen et al. 2009). Newer data suggests an increased rate of acute myocardial infarction in patients with bipolar disorder, with an increased risk in patients diagnosed with bipolar disorder as compared to patients with schizophrenia, as well as an increased relative mortality rate in patients with bipolar disorder as compared to patients with schizophrenia (Bodén et al. 2015; Wu et al. 2015). Despite the known increased rate of mortality in patients with SMI relative to the general population as caused by cardiovascular disease, adequate treatment of cardiovascular risk factors in these patients are often less likely to occur as compared to the patients with cardiovascular disease without SMI (Kilbourne et al. 2008). Moreover, data suggests medical conditions such as hypertension, dyslipidemia, and diabetes being more frequently present but not previously diagnosed in patients with SMI admitted to hospital as compared to controls (Briskman et al. 2012). The lack of prophylactic treatment, or factors related to the bipolar disorder itself results in an increased rate of pulmonary embolism (Strudsholm et al. 2005) and an increased rate of stroke (Wu et al. 2013; Prieto et al. 2014), as compared to the general population. Furthermore, data on intravenous thrombolysis in the treatment of stroke shows that patients with SMI are frequently less treated than patients not diagnosed with a psychiatric disorder (Bongiorno et al. 2018).

A shared link in pathophysiology between bipolar disorder and diabetes has been proposed (Calkin et al. 2013), with common genetic links, epigenetic factors as well as lifestyle and treatment of bipolar disorder being factors associated with a poorer glycemic control and a worse general outcome of diabetes and cardiovascular disease as a whole (Calkin et al. 2013). Furthermore, a reduced cerebrovascular reactivity among adolescents with bipolar disorder compared to psychiatrically healthy controls has been observed, suggesting a poorer cerebrovascular health (Urback et al. 2018).

## Respiratory disease

Data on respiratory disease in patients with SMI is scarcer as compared to data on cardiovascular morbidity and mortality, but respiratory symptoms are frequently described by patients (Burke et al. 2018). A doubled prevalence of approximately 6% as well as an almost doubled incidence of diagnosed chronic obstructive pulmonary disease (COPD) have been described in patients with bipolar disorder as compared to the general population (Hsu et al. 2017). Similar to the data on cardiovascular disease, the link between COPD and bipolar disorder is bidirectional with a 40% increased incidence of bipolar disorder in patients with COPD as compared to those not diagnosed (Tsai et al. 2016; Su et al. 2017). Similar to COPD, a link between asthma and bipolar disorder has been shown, with an increased incidence of bipolar disorder in patients with asthma, and with prednisolone treatment further increasing the risk of a bipolar diagnosis (Lin et al. 2014), as well as asthma being more common in patients diagnosed with bipolar disorder than in controls (Patten and Williams 2007; Perugi et al. 2015).

Despite these findings, the treatment effects on COPD or asthma in patients diagnosed with bipolar disorder or any other SMI diagnosis have not yet been investigated.

## Cancer

Studies have shown both an increased and no increased incidence of cancer in patients diagnosed with bipolar disorder as compared to the general population (McGinty et al. 2012; Martinsson et al. 2016). Some data suggests an increased incidence of cancers in the digestive tract, in the respiratory system and in the endocrine glands in patients with bipolar disorder (Martinsson et al. 2016), whereas other data shows an increased risk only in males with bipolar disorder (Lin et al. 2013). The differences in incidence could be a result of differences in study populations as well as differences in the follow-up time in studies. Cancer most often is diagnosed after the age of 60 years, which could bias data due to the generally increased mortality rate in patients with bipolar disorder resulting in patients possibly dying due to other causes before a cancer could be diagnosed (Howard et al. 2010; Guan et al. 2013).

Compared to the general population, patients with SMI have an increased mortality after cancer diagnosis, independent of cancer stage (Manderbacka et al. 2017; Toender et al. 2018). Strikingly, no increased risk of advanced cancers at diagnosis has been observed in patients diagnosed with SMI as compared to the general population (Chang et al. 2014), suggesting that differences in survival may be due to treatment and course after cancer diagnosis and not due to a delayed diagnosis. Studies investigating the treatment of cancers in patients with mental illness have in fact shown a reduced chance of surgery for patients with oral cancer with or without adjuvant therapy, and patients with mental illness had an increased mortality rate as compared to controls (Chang et al. 2013). Similar findings were seen in patients with a diagnosed mood disorder and breast cancer in which patients with a mood disorder had an overall poorer outcome, albeit patients diagnosed with bipolar disorder did not have an increased mortality rate (Kanani et al. 2016). Elderly patients diagnosed with mental disorders showed a delay in initiation of cancer treatment, perhaps explaining some of the differences in mortality between patients with mental illness and the general population (Iglay et al. 2017a), and highlighting the possible need for coordinated treatment to ensure similar treatment outcomes and options in patients with SMI as compared to patients without a psychiatric disorder (Cole and Padmanabhan 2012; Iglay et al. 2017b).

## Discussion

Patients with bipolar disorder have an increased mortality rate relative to the general population, and the main causes of death are somatic diseases (Hayes et al. 2017; John et al. 2018; Staudt Hansen et al. 2018). The underlying factors resulting in an increased mortality rate in patients with bipolar disorder and SMI in general are not fully elucidated, but concerns regarding disparity in diagnosis of risk factors, primary prophylactic intervention, secondary prophylactic efforts and treatment have been raised (Mitchell et al. 2014) although direct links to psychiatric related factors like genetic load, treatment or non-acceptance of offered treatment, cannot be ruled out.

Patients with bipolar disorder may not be offered diagnostic procedures or treatment for somatic diseases simply because of the psychiatric diagnosis per se or because they are unable to follow current treatment regiments due to the disorder. In general, there is data supporting a less positive attitude towards patients with SMI resulting in poor communication, disregard for patients’ dignity and delays in investigation or treatment in the physical treatment setting (Happell et al. 2012; Neupane et al. 2016; Noblett et al. 2017), as well as in the community (Mirnezami et al. 2016). Several experts have proposed the initiation of coordinated or combined treatment options with collaboration between specialized psychiatric and somatic care for patients with SMI to increase the likelihood of optimal treatment as well as reducing attrition rates of patients treated (Fleischhacker et al. 2008; Nielsen and Licht 2018). The increased collaborative treatment options proposed have also been endorsed by associations and societies associated with mental health, diabetes and cardiology (De Hert et al. 2009).

In general, there is a lack of investment in primary treatment of bipolar disorder as compared to other severe mental disorders, and especially as compared to somatic diseases (MacEwan et al. 2016). This might also be driving the increased mortality rate in patients with bipolar disorder, as well as other patients diagnosed with SMI, since patients with insufficiently treated SMI may be less likely to seek help for somatic illness and less likely to adhere to relevant treatments for these illnesses. On the other hand, the current available psychopharmacological treatments for SMI have been shown to increase the rate and severity of multiple somatic disorders including cardiovascular and respiratory diseases (Correll et al. 2015; Wu et al. 2018), but they seem to reduce the risk of intentional self-harm as cause of death (Khan et al. 2013). Generally, there is a lack of data on effects for somatic illnesses in patients with SMI, and the field seems generally poorly prioritized in terms of research.

In patients with bipolar disorder admitted due to somatic illness, a more severe course of the somatic disease has been observed, and more co-morbidities diagnosed, as compared to age- and gender-matched controls also admitted (Schoepf and Heun 2014). These findings could be interpreted as patients with bipolar disorder being susceptible to more severe somatic disease, which might not be modifiable, but could also be a result of a lack of primary and secondary prophylaxis, as well as a lack of sufficient treatment of diagnosed somatic morbidities. Previous data in patients with SMI have shown a need for intensified treatment to remove the differences in mortality rates after myocardial infarction, showing increasing mortality rates with decreasing treatment intensity when compared to the general population (Kugathasan et al. 2018a, b). Furthermore, data supports a lowered use of treatment for cardiovascular disease including thrombolytics for stroke (Bongiorno et al. 2018) and lower rates of cardiovascular prophylactic treatment (Laursen et al. 2009, 2014). Lastly, data on cancer show a similar stage when diagnosed, but a reduced likelihood of receiving surgery and adjuvant treatment, and shows a poorer survival as compared to patients not diagnosed with SMI (Chang et al. 2013, 2014; Manderbacka et al. 2017; Toender et al. 2018).

Treatment for somatic disease has significantly improved over the last decades with implementation of improved diagnostics, better primary and secondary prophylactic treatment options, as well as a general reduction in lifestyle behavior associated with somatic disease (Roth et al. 2015; Korhonen et al. 2017). During the same time period, an increasing excess mortality relative to the general population has been seen in patients with bipolar disorder (Hayes et al. 2017; Staudt Hansen et al. 2018), perhaps suggesting a lack of benefit in the population of patients with SMI of the general improvements in somatic treatments (Armstrong et al. 1999; Laursen and Nordentoft 2011; Lautrup et al. 2018; Kugathasan et al. 2018a, b; Degett et al. 2019).

## Conclusion

Patients with bipolar disorder have an excess mortality with somatic diseases most often being defined as cause of death. The current management of somatic illness occurring in patients with bipolar disorder has not been able to decrease the mortality gap between the general populations and patients with bipolar disorder, and the underlying factors could be both patient as well as treatment setting related. Data has shown an increased survival in the general population, most likely due to improvements in treatments as well as prophylaxis, but these interventions are used less often in patients with bipolar disorder and SMI in general, perhaps, at least in part, as a result of negative attitudes towards patients with SMI. Collaborative care established between psychiatric and somatic treatment facilities might reduce the treatment gap, the mortality gap as well as diminish the possible negative attitudes towards somatic treatment of patients with SMI.
